# Hydrophobic Optimization of Functional Poly(TPAE-co-suberoyl chloride) for Extrahepatic mRNA Delivery following Intravenous Administration †

**DOI:** 10.3390/pharmaceutics13111914

**Published:** 2021-11-12

**Authors:** Xueliang Yu, Shuai Liu, Qiang Cheng, Sang M. Lee, Tuo Wei, Di Zhang, Lukas Farbiak, Lindsay T. Johnson, Xu Wang, Daniel John Siegwart

**Affiliations:** Department of Biochemistry, Simmons Comprehensive Cancer Center, The University of Texas Southwestern Medical Center, Dallas, TX 75390, USA; Xueliang.Yu@UTSouthwestern.edu (X.Y.); Shuai.Liu@UTSouthwestern.edu (S.L.); Qiang.Cheng@UTSouthwestern.edu (Q.C.); SangM.Lee@UTSouthwestern.edu (S.M.L.); Tuo.Wei@UTSouthwestern.edu (T.W.); Di.Zhang@UTSouthwestern.edu (D.Z.); Lukas.Farbiak@UTSouthwestern.edu (L.F.); Lindsay.Johnson@UTSouthwestern.edu (L.T.J.); Xu.Wang@UTSouthwestern.edu (X.W.)

**Keywords:** polyesters, nanoparticles, polyplex, mRNA delivery, luciferase mRNA, Cre recombinase mRNA

## Abstract

Messenger RNA (mRNA) has generated great attention due to its broad potential therapeutic applications, including vaccines, protein replacement therapy, and immunotherapy. Compared to other nucleic acids (e.g., siRNA and pDNA), there are more opportunities to improve the delivery efficacy of mRNA through systematic optimization. In this report, we studied a high-throughput library of 1200 functional polyesters for systemic mRNA delivery. We focused on the chemical investigation of hydrophobic optimization as a method to adjust mRNA polyplex stability, diameter, pKa, and efficacy. Focusing on a region of the library heatmap (PE4K-A17), we further explored the delivery of luciferase mRNA to IGROV1 ovarian cancer cells in vitro and to C57BL/6 mice in vivo following intravenous administration. PE4K-A17-0.2C8 was identified as an efficacious carrier for delivering mRNA to mouse lungs. The delivery selectivity between organs (lungs versus spleen) was found to be tunable through chemical modification of polyesters (both alkyl chain length and molar ratio in the formulation). Cre recombinase mRNA was delivered to the Lox-stop-lox tdTomato mouse model to study potential application in gene editing. Overall, we identified a series of polymer-mRNA polyplexes stabilized with Pluronic F-127 for safe and effective delivery to mouse lungs and spleens. Structure–activity relationships between alkyl side chains and in vivo delivery were elucidated, which may be informative for the continued development of polymer-based mRNA delivery.

## 1. Introduction

Messenger RNA (mRNA) holds great promise for continued therapeutic applications, including vaccines, protein replacement therapy, and immunotherapy [1,2,3,4,5,6,7,8,9,10,11,12,13,14]. The recent success of mRNA lipid nanoparticle (LNP) vaccines for SARS-CoV-2 has solidified the significant impact of this life-saving approach [15,16]. Delivery of mRNA also provides a reliable approach for emerging genome editing technologies, including the clustered regularly interspaced short palindromic repeat (CRISPR)-associated protein (Cas) (CRISPR/Cas) because of its effective and controllable expression of gene editing proteins, both in vitro and in vivo [17,18,19,20,21,22,23,24,25,26]. Since mRNA does not integrate into the host’s genome and results in transient protein expression, this approach may limit the risk of off target mutations in cells. Due to the unique structure of mRNA (single-stranded; typically more than 1000 nucleotides) [27], stable carriers are required for protecting mRNA from degradation and facilitating intracellular delivery [28]. Katalin Karikó, Drew Weissman, and colleagues discovered that incorporation of modified nucleobases can significantly reduce the immunogenicity of exogenously introduced mRNA [29,30,31,32]. This important discovery has greatly influenced the development of mRNA therapeutics, most recently and significantly through the incorporation of modified nucleosides in the COVID-19 mRNA vaccines that have been administered to hundreds of millions of people worldwide. The great progress of pDNA and siRNA delivery [33,34,35,36,37,38,39,40], coupled to progress in understanding the fundamental science behind mRNA [41,42,43], has established a strong foundation to further expand the development of improved mRNA carriers.

In addition to improving safety and efficacy, mRNA delivery to extrahepatic targets remains challenging. We recently reported an approach called Selective ORgan Targeting (SORT) [22,26,44,45] that can engineer LNPs for delivery of a variety of RNAs, proteins, and ribonucleoprotein complexes to specific organs by introducing an additional SORT molecule that enables programmable delivery to specific tissues including the lung, spleen, and liver. Additional exciting reports have demonstrated mRNA delivery to the lungs, spleen, bone marrow, and T cells, although the mechanism of action remains unclear [46,47,48,49]. These efforts have been limited to low-molecular weight lipids used to form multi-component LNPs that do allow much flexibility for tuning the chemistry of the ionizable amino lipid without reducing efficacy. In contrast, polymers offer a much greater degree of chemical diversity of functional groups and physical properties. It is much easier to tune the chemical structures and properties of polymers without affecting activity for gene delivery. Polymers additionally have some potential further advantages with respect to scalability and production in large batches. Although polymer-mediated mRNA delivery to organs such as the lungs and spleen [50,51] has been demonstrated, there remains significant opportunities for further chemical exploration and determination of structure–activity relationships (SAR) to guide the development and understanding of mRNA carriers.

Cationic polymers have been extensively explored for pDNA delivery, which offers guidance for use in mRNA delivery. For example, polyethylenimine (PEI)-based polymers can deliver luciferase mRNA into cells [52]. However, in order to be used in clinical research, polymer-based carriers should exhibit low toxicity and facile degradability to improve safety. Poly(β-amino ester)s (PBAEs) [50,51,53] and charge-altering releasable transporters (CARTs) [49,54,55] are additional classes of polymers capable of delivering mRNA both in vitro and in vivo. Anderson and co-workers reported degradable polymer–lipid hybrid nanoparticles for systemic mRNA delivery to the lungs. In 2019, the same research group reported a different administration method though inhalation to the lungs using a hyperbranched poly(β-amino esters) (hPABEs). In 2017, Wender and co-workers synthesized a new type of oligo(carbonate-*b*-α-aminoester) termed charge-altering releasable transporters (CARTs) [55,56,57] that enabled mRNA delivery to lymphocytes. Overall, it is evident that the delivery efficacy, safety, and tissue targeting of polymer-mRNA delivery systems can be further optimized.

Recently, our lab developed a facile and scalable method for the synthesis of polyesters with functional -ene side chains via the condensation of diacyl chlorides and trimethylolpropane in the presence of an organic base [58]. Previously, this platform showed activity for siRNA and mRNA delivery in vitro and in vivo [38,39,41]. Some functional polyester nanoparticles showed selectivity for certain non-small cell lung cancer (NSCLC) cells, which fully relied on the physiochemical properties of the polyesters themselves to enable selective cell uptake [38]. Cellular uptake studies have indicated that polyplex internalization is dominated by clathrin-dependent endocytosis. This indicated that precise changes in chemical composition can greatly alter cell (and potentially organ)-level targeting. These efforts led to identification of multiple lead polymers, including PE8K-A17-0.2C6, PE4K-A13-0.33C6, PE4K-A13-0.33C10, and PE4K-A17-0.33C12. The hydrophobic domains of polymer backbones and side chains, as well as small molecule lipids [59], play a crucial role in amphipathic nanoparticle self-assembly formation [43,60,61,62]. Therefore, the goal of this manuscript is to focus on further optimizing the hydrophobic motif of polymers for delivery to the lungs and spleen.

In order to address the issues of low delivery efficacy and toxicity of cationic polymers, various chemical modifications have been explored [63,64]. Extensive research [64] has shown that hydrophobic modifications on polymers such as PEI, chitosan, poly(L-lysine) and poly(2-*N*-(dimethylaminoethyl) methacrylate) (pDMAMA) can have a significant effect on gene delivery by increasing the physical encapsulation of nucleic acids, enhancing cellular uptake and improving serum stability. We hypothesized that the hydrophobic domains of functional polyester backbones can also modulate mRNA delivery. In this paper, we focus on the hydrophobic side chain modification of polyesters to optimize polymer-based mRNA delivery and establish SAR. Via the high-throughput screening of 1200 functional polyesters, we were able to identify superior polymeric carriers for in vivo mRNA delivery. The delivery selectivity between organs (lungs versus spleen) was found to be tunable through modifying the side chain alkyl chain length and formulation conditions. Cre recombinase mRNA targeting the Lox-stop-lox tdTomato sequence in a mouse model was delivered to establish proof-of-concept gene editing. We also elucidated structure–activity relationships between alkyl side chains and in vivo delivery efficacy. We further demonstrate that hydrophobic modifications of cationic polymers could be highly beneficial for mRNA delivery. This work contributes to the overall body of literature on mRNA delivery carriers and further validates that mRNA therapeutics are an important area of research that may continue to yield next-generation vaccines and therapeutics.

## 2. Materials and Methods

### 2.1. Ethics Statement

All animal experiments were approved by the Institutional Animal Care and Use Committee (IACUC) of the University of Texas Southwestern Medical Center and were consistent with local, state, and federal regulations, as applicable.

### 2.2. Materials

All chemicals (amines, alkyl thiols, and solvents) for the synthesis of functional polyesters were purchased from Sigma-Aldrich (Burlington, MA, USA), TCI America (Portland, OR, USA), or Fisher Scientific (Hampton, NH, USA). Luciferase and Cre mRNA were obtained from TriLink Biotechnologies (San Diego, CA, USA). RPMI-1640, fetal bovine serum (FBS), phosphate buffered saline (PBS), and PEO101−PPO56−PEO101 (Pluronic F-127, Mw = 12 600, PDI = 1.05) were purchased from Sigma-Aldrich. DMG-PEG lipid (Sunbright GM-020) was purchased from NOF America (White Plains, NY, USA). The Quant-iT RiboGreen RNA assay kit was obtained from Life Technologies (Carlsbad, CA, USA). The ONE-Glo + Tox luciferase assay kit was obtained from Promega (Madison, WI, USA).

### 2.3. Preparation and Characterization of mRNA Nanoparticles

The different molecular weights of ene-bearing polyesters were synthesized according to previously reported protocols [38,39,41,58]. The library of 1200 functional polyesters was synthesized through thiol–ene reaction under UV. For in vitro studies, mRNA NPs were prepared by adding diluted functional polymers (3 g/L in DMSO) into mRNA buffer solution (citric acid/trisodium citrate buffer, pH 4.2, 10 mM) at a polyester/mRNA ratio of 30:1 (*wt/wt*) and a final mRNA concentration of 1.25 ng/μL. For in vivo studies, 5 wt% Pluronic F-127 was added to the functional polyester DMSO solution, which was then diluted with EtOH (DMSO: EtOH = 1:2, *v/v*). An mRNA buffer solution (citric acid/trisodium citrate buffer, pH 4.2, 10 mM) was added to the above solution (aqueous: organic = 3:1, *v/v*) by hand-mixing to form the polyplex nanoparticles. The polyplex nanoparticles were dialyzed against PBS (1X) for 2h before injection to mice by I.V. administration. The size, polydispersity index (PDI), and zeta potential of the polyplex nanoparticles were measured using a Zetasizer Nano ZS (Malvern, He−Ne, λ = 632 nm). mRNA binding was tested by utilizing the Quant-iT RiboGreen RNA assay kit. The global/apparent LNP pKa was determined by the TNS assay [26,65].

### 2.4. In Vitro Delivery of mRNA Polyplex Nanoparticles

RPMI-1640 medium with 5% FBS and 1% Penicillin/Streptomycin (P/S) was used to culture IGROV1 ovarian cancer cells. IGROV1 cells were seeded into opaque white 96-well plates (with a density of 10,000 cells/well) and incubated for 24 h at 37 °C and 5% CO_2_ in a humidified atmosphere. After 24 h, the old medium was replaced with fresh RPMI-1640 medium with 5% FBS and 1% Penicillin/Streptomycin (P/S) (200 μL/well) followed by the addition of 20 μL of mRNA polyplex nanoparticles (25 ng mRNA/well). The final mixture was incubated for 24 h before testing the cell viability and luciferase expression by using ONE-Glo + Tox luciferase assay kits. All transfection assays were performed in triplicate, and the average with standard deviation was reported.

### 2.5. In Vivo Delivery of mRNA Polyplex Nanoparticles

C57BL/6 mice were purchased from Charles-River. For Luc mRNA delivery, polyplex nanoparticles with mRNA were prepared as described above. 200 μL of mRNA polyplex NPs (10 μg of Luc mRNA, 0.5 mg/kg) were administered to C57BL/6 mice (18–24 g) by tail vein injection. After 6 h, D-luciferin (150 mg/kg) was injected via I.P. administration. After 5 min, whole body and ex vivo organs of mice were imaged by an IVIS Lumina imaging system. For the tdTomato mice (Ai9) experiments, 200 μL of mRNA polyplex NPs (10 μg of Cre mRNA, 0.5 mg/kg) was administered to tdTomato mice (18–24 g) by tail vein. After 2 days, the mice were sacrificed, and ex vivo organs of mice were imaged by an IVIS Lumina imaging system. For biodistribution, 200 μL of mRNA polyplex NPs (10 μg of Cy5-mRNA, 0.5 mg/kg) was administered to C57BL/6 mice (18–24 g) by tail vein. After 6 h, the mice were sacrificed, and the organs of mice were imaged ex vivo by an IVIS Lumina imaging system.

### 2.6. Statistical Analysis

All data are presented as the mean ± SD unless otherwise indicated. Statistical analyses were performed using GraphPad Prism version 9 (GraphPad Software). One-tailed unpaired Student’s t-test was used to determine the significance of the indicated comparisons. *p*-values < 0.05 (*), *p* < 0.01 (**), *p* < 0.001 (***) and *p* < 0.0001 (****) were considered to be statistically significant.

## 3. Results and Discussion

**Figure 1:**
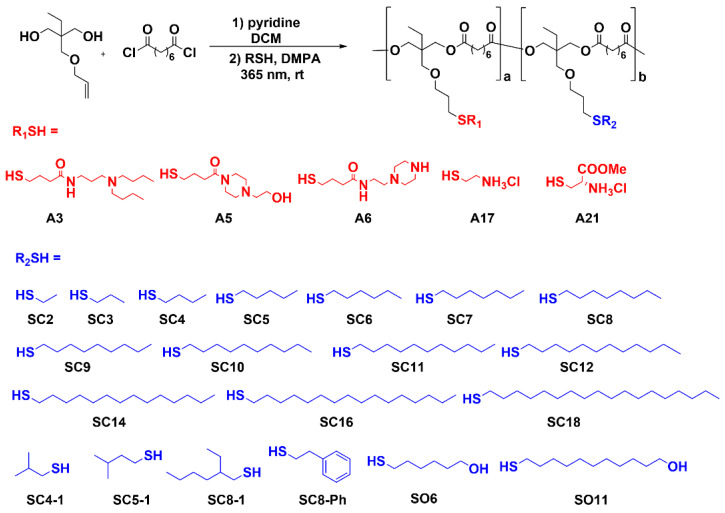
A combinatorial library of functional polyester NPs was screened in IGROV1 cells to optimize mRNA delivery materials. A library of 1200 functional polyesters was chemically synthesized for the screening of mRNA delivery. Polyesters were modified with amino thiols (R_1_SH) and alkyl thiols (R_2_SH) to generate a combinatorial polymer library. Amino thiols are named **A** followed by a number; alkyl thiols are named **SC** or **SO** followed by the number of carbons. Functional polyesters with *Mw* 4200 g/mol (PE4K), 8300 g/mol (PE8K), and 17,000 g/mol (PE17K) were modified with five amino thiols (**A3**, **A5**, **A6**, **A17**, and **A21**) and all 20 alkyl thiols at SC:A molar feed ratios of 1:4, 1:2, 1:1, and 2:1. Functionalized polymers are named by the polyester *Mw*, amino modification, and the mole fraction of alkyl modification. All functional polyesters were examined for in vitro mRNA delivery efficacy. Selected functional polyesters (0.2C4 to 0.2C11; 0.3C5 to 0.3C9) were examined for in vivo mRNA delivery efficacy.

High-throughput synthesis and screening is an established approach for the discovery of effective carriers for delivery of nucleic acids [33]. Hydrophobic modification plays a key role in improving the efficacy and reducing the toxicity of polymers for nucleic acid delivery. Due to the amphiphilic lipid composition of plasma and endosome membranes, increasing the hydrophobicity of polymer carriers could increase polyplex cellular uptake and endosomal escape [64,66]. For example, the Forrest group reported that acetylated PEI can increase transfection efficiency by up to 58-fold compared to unmodified PEI [67,68]. Few studies have shown that hydrophobic modification can improve mRNA delivery. Here, we built a library of 1200 functional polyesters with different functional groups (alkyl- and amino-) utilizing our previous polycondensation method [58], and used in vitro/in vivo screening to identify vehicles for mRNA delivery. The library design is depicted in Figure 1. As hydrophobic modification can change the delivery efficacy [64,66,67,68], here, we aimed to expand the chemical diversity of the hydrophobic motif. We used different categories of alkyl thiols, such as linear (**SC2** to **SC18**), branched (**SC4-1**, **SC5-1**, and **SC8-1**), aromatic (**SC8-Ph**), and hydroxyl group containing (**SO6** and **SO11**), to maximize the diversity. Whitehead et al. [43]. reported that the branched-tail ionizable cationic lipid can enhance the delivery efficacy of mRNA compared to the related linear lipid due to enhanced ionization at endosomal pH. With respect to the ionizable amine-containing side chains, we chose four different amines (**A3**, **A5**, **A6**, and **A17**), which have been proven effective in the delivery of either siRNA or mRNA [38,39,41]. Based on the chemical structures of these previously identified lead domains, we included a new amine, **A21**, due to its similarity to **A17** and the fact that it is an amino acid (cysteine) derivative, as the amine component. Three different molecular weights of polyesters were chosen as the backbone for the thiol–ene reaction under UV conditions in order to study MW effects.

**Figure 2:**
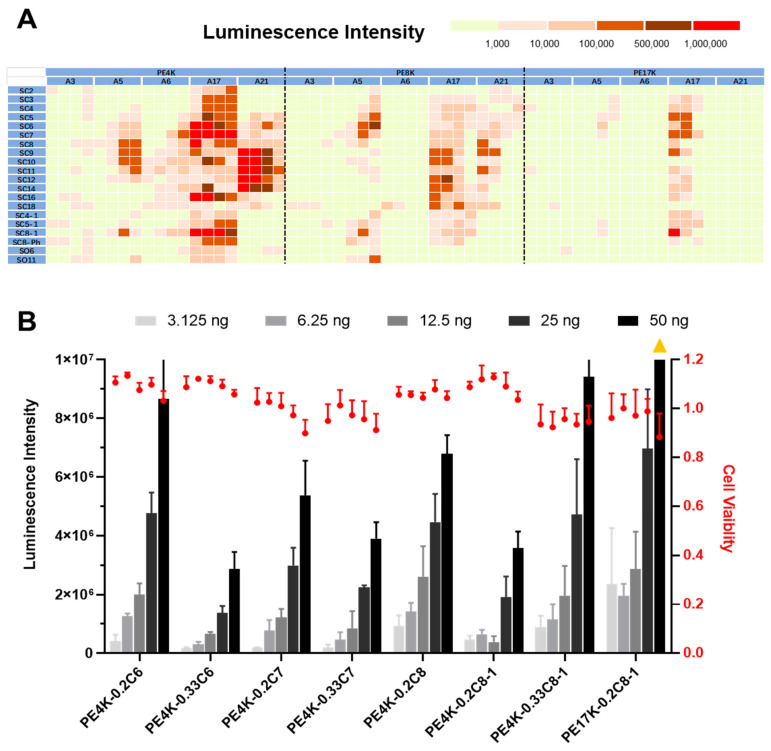
(**A**) Heatmap of in vitro screening of functional polyesters for luciferase mRNA (Luc mRNA) delivery to IGROV1 cells enabled identification of efficacious mRNA carriers. Each sub-column of corresponding amino thiols represent the SC:A molar feed ratios of 1:4, 1:2, 1:1, and 2:1. The Luc mRNA dose was 25 ng mRNA/well (181 pM). (**B**) Selected functional polyesters for Luc mRNA delivery efficacy and toxicity with dose response (mean ± s.d., *n* = 3; triangle symbol means over the range; all polyesters in B are **A17**-modified).

The results of in vitro studies are shown in Figure 2 (also see Table S1, Supporting Information). mRNA polyplexes with lower molecular weight (PE4k) functional polyesters were able to deliver luciferase (Luc) mRNA into IGROV1 cells more efficiently than the corresponding functional polyesters prepared from higher MW precursor polymers in general. These mRNA delivery results are in agreement with previous studies using related functional polyesters for siRNA delivery, suggesting that a balance between the polymer MW and hydrophobicity relating to physical chain entanglement and intermolecular forces may be important for delivery efficacy [38,69,70,71]. When further analyzing the results, the **A17** (cysteamine)-modified polyesters again emerged as the most active region, which confirmed our earlier results in siRNA and mRNA delivery studies. As the current paper focuses on hydrophobic modifications, it was interesting to identify more effective materials (**SC6, SC7, SC8,** and **SC8-1** modified polyesters) than those that have been previously identified. Notably, one of the high-molecular weight polyesters (PE17K-A17-0.2C8-1) also possessed great in vitro delivery efficacy. These results confirmed that hydrophobic optimization can improve mRNA delivery efficacy of polyplex carriers. With further respect to the hydrophobic domains, the linear alkyl-modified polyesters were slightly superior over branched alkyl-modified polyesters (**SC4-1** versus **SC4**; **SC5-1** versus **SC5**), with the exception of the eight-carbon alkyl-modified ones. These results are in contrast to recent observations of branching in small molecule lipid designs [43]. Alkyl lengths that were too short (**SC2**) or too long (**SC18**) did not show in vitro delivery efficacy, which has been previously observed in lipid designs [40,43]. The terminal hydroxyl alkyl-modified polyesters (**SO6** and **SO11**) did not show great delivery efficacy in vitro, which could potentially be due to the increasing hydrophilicity of the extra hydroxyl group destabilizing the mRNA-polyplex self-assembly.

**Figure 3:**
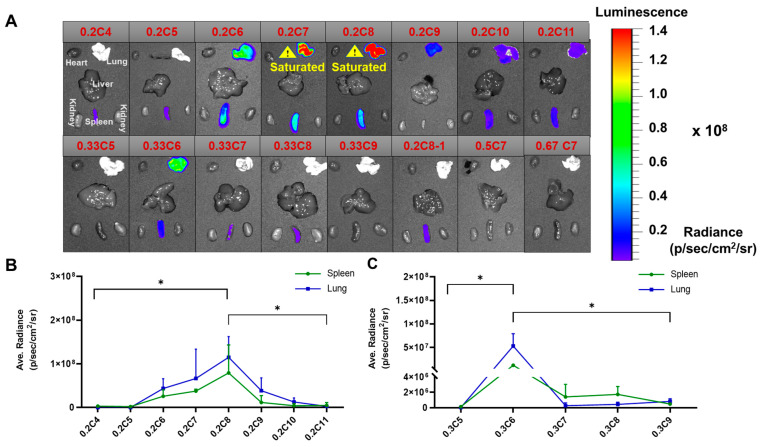
In vivo screening of Luc mRNA delivery. Top functionalized polyesters were examined for their ability to deliver Luc mRNA in vivo. C57BL/6 mice were randomly grouped and intravenously injected with 0.5 mg/kg Luc mRNA (*n* = 2). Luminescence was quantified 6 h after injection. (**A**) Ex vivo bioluminescence images of selected polyesters. The yellow triangle denotes detector saturation of signals. (**B**,**C**) The average luminescence for the spleen and lungs was plotted. The parent polyester was PE4K-A17 for all the above selected functionalized polyesters (mean ± s.d., *n* = 2, *p* < 0.05 (*)).

Based on the in vitro results, we chose the PE4K-A17 sub-group materials to further test the delivery efficacy in vivo. Previously, we identified that the addition of 5 weight% of Pluronic F127 was a crucial surface coating component to stabilize the polyplex nanoparticles for intravenous administration [41]. The in vivo results (Figure 3) demonstrated that luciferase expression changed between organs (lungs and spleen) with the different alkyl chains lengths and molar ratios. When the SC:A molar feed ratio equaled 1:4 (0.2C), eight carbon alkyl chains (SC8) yielded the best performance. Interestingly, when the SC:A molar feed ratio was increased to 1:2 (0.33C), a shorter alkyl chain (six carbon, SC6) showed the highest in vivo efficacy. Overall, PE4K-A17-0.2C8 produced the best mRNA delivery efficacy. Interestingly, the delivery efficacy of linear (**SC8**) functional polyester was much better than branched (**SC8-1**) functional polyesters in the lungs, and the organ selectivity was reversed. We concluded that both the alkyl chain length and molar ratio used in the formulation played roles in delivery efficiency and organ selectivity. Short alkyl chains (**SC4**, **SC5**) and higher molar feed ratio of alkyl chains (0.33C7 and 0.33C8) favored the spleen, but the overall delivery efficacy was sensitive to these parameters. 0.5C7 and 0.67C7-modified polyesters (PE4K-A17) were unable to successfully deliver mRNA in vivo.

**Figure 4:**
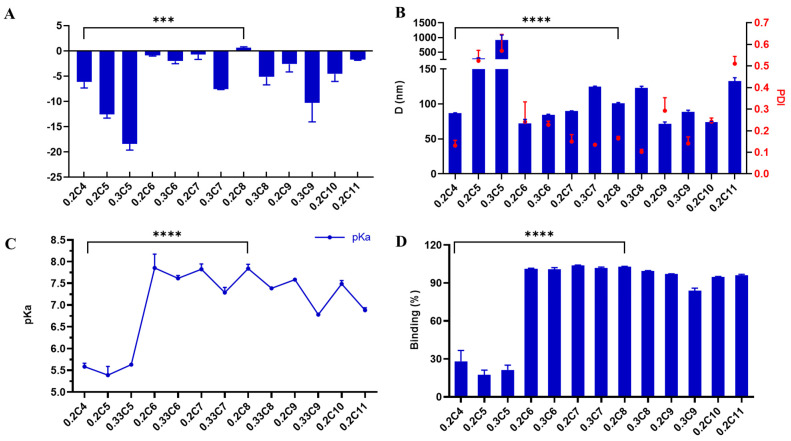
Physicochemical properties of mRNA polyplex nanoparticles with selected functional polyesters (PE4K–A17): (**A**) surface charge; (**B**) PDI and size; (**C**) pKa; (**D**) RNA binding (mean ± s.d., *n* = 3, *p* < 0.001 (***) and *p* < 0.0001 (****)).

**Figure 5:**
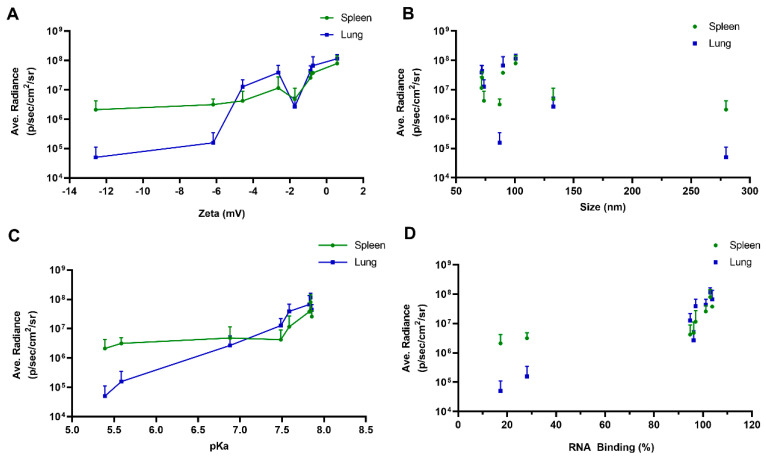
Correlations between ex vivo luminescence intensity and physicochemical properties of mRNA polyplex. (**A**) Surface charge versus luminescence intensity. (**B**) Size versus luminescence intensity. (**C**) pKa versus luminescence intensity. (**D**) RNA binding versus luminescence intensity (mean ± s.d., *n* = 3).

In our previous report, the chemical properties of functional polyesters could enable selective delivery to patient-matched cancer cells over normal cells [38]. Other reports have further correlated physical properties to in vivo delivery efficacy [43]. Next, we measured the physical properties of nanoparticles to determine SARs (Figure 4 and Figure 5). Most selected polymers were able to bind to mRNA tightly (>80%) and form controlled polyplex nanoparticles with diameters < 150 nm, except for SC4- and SC5-modified polyesters. The short alkyl chains (**SC4** and **SC5**) have less hydrophobicity, causing the nanoparticles to be less stable (large size and large PDI). These poor physical properties may explain the low in vivo delivery efficacy of SC4- and SC5-modified functional polyesters. In Figure 5, the correlations between ex vivo luminescence intensity and the physicochemical properties of mRNA polyplex nanoparticles (0.2C4 to 0.2C11) are plotted. The surface charge of mRNA polyplex nanoparticles showed positive correlations to ex vivo luminescence intensity for both organs (lungs and spleen). PE4K-A17-0.2C6, 0.2C7 and 0.2C8 have a surface charge close to neutral, which may benefit in vivo delivery by improving stability and reducing MPS clearance. Consistent with our own and other studies on polymer-mediated mRNA delivery [41,50,53,55], mRNA translation to protein was mainly observed in the lungs and spleen. However, the biodistribution results tracking Cy5-mRNA showed that most polyplex nanoparticles accumulate in the liver (Figure S1). This has also been observed for other polymeric mRNA carriers [72]. Therefore, it will be useful in future studies to determine the probable complex mechanism of this behavior, wherein the organ accumulation of mRNA delivery systems including lipid- and polymer-based carriers do not always lead to successful mRNA translation to protein. These observations also offer the opportunity to design liver-targeted mRNA polyplexes in the future, which are currently lacking for polymer-based systems. Although the mechanism remains unclear, PE4K-A17-0.2C8 accumulated in the lungs, which verifies the lung activity and potential superiority of PE4K-A17-0.2C8 polyplex nanoparticles over other tested polymers.

**Figure 6:**
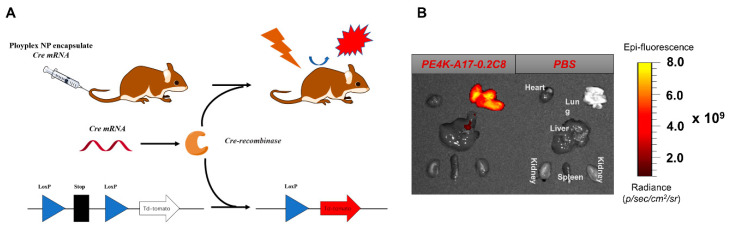
(**A**) TdTomato mouse model representation. (**B**) After I.V. injection of polyplex nanoparticles at a dosage of 0.5 mg/kg, tdTomato fluorescence was detected in lungs though ex vivo imaging.

To assess additional applications of this carrier, we utilized a tdTomato mouse model, which contains a Lox-Stop-Lox tdTomato cassette in all cells, to test its gene editing capability via the deployment of Cre recombinase mRNA (Cre mRNA). Following translation of Cre mRNA to Cre protein and deletion the of stop codons, cells will express red fluorescent tdTomato protein and be readily detectable [73,74,75]. We formulated Cre mRNA into nanoparticles and then injected NPs into mice via I.V. administration at a dosage of 0.5 mg/kg (Figure 6). Clear tdTomato signal throughout the lungs was observed by ex vivo lung imaging. It will be valuable in the future to understand which cell type(s) are transfected in order to match capabilities with therapeutic applications [41,50]. The results indicate that this carrier has potential applications in the deployment of proteins for gene editing for targets in the lungs due to the successful activation of tdTomato [76,77,78].

## 4. Conclusions

In this paper, we synthesized a combinatorial library of functional polyesters with a focus on hydrophobic optimization to identify efficacious materials for mRNA delivery by high-throughput screening. Following in vitro screening, we further examined a sub-portion of the library (PE4K-A17), which exhibited high delivery efficacy of Luc mRNA NPs in IGROV1 ovarian cancer cells. The delivery efficacy in vivo was examined by IV injection of formulated mRNA polyplex nanoparticles with 5% (*wt*/*wt*) of Pluronic F-127 into mice. PE4K-A17-0.2C8 was identified as the optimal polymeric carrier for the delivery of mRNA into mouse lungs. The delivery selectivity between organs (lungs versus spleen) was found to be tunable through chemical modification of polyesters (both alkyl chain length and molar ratio in formulation). Finally, we employed a tdTomato mouse model to demonstrate that this efficient mRNA delivery system could potentially be used to treat genetic lung diseases.

## Data Availability

Not applicable.

## Supplementary Materials

The following are available online at https://www.mdpi.com/article/10.3390/pharmaceutics13111914/s1, Figure S1: Imaging of mice organs after injection of Cy-5 mRNA-loaded functional polyesters; Figure S2: Selected NMR spectra of polyester and functional polyester; Figure S3: GPC (THF) curves of polyesters; Table S1: Raw data of in vitro screening of functional polyesters for luciferase mRNA delivery.

## Dedication

The authors dedicate this article to honor Katalin Karikó for her pioneering research in nucleoside modified mRNA. Katalin Karikó’s discoveries directly contributed to the development of safe and effective mRNA vaccines against the SARS-CoV-2 virus, which causes the COVID-19 disease. Her discoveries have made a tremendously positive impact on science, health, and human society.
