# Supplementary material for: Hydrophobic Optimization of Functional Poly(TPAE-co-suberoyl chloride) for Extrahepatic mRNA Delivery following Intravenous Administration
Source: Pharmaceutics. 2021 Nov 12;13(11):1914. doi: 10.3390/pharmaceutics13111914 (PMC8624493; doi:10.3390/pharmaceutics13111914)
Supplement: Supplementary file 1 [file pharmaceutics-13-01914-s001.zip › pharmaceutics-1344360-supplementary.pdf]

Supplementary materials: Hydrophobic Optimization of Functional poly (TPAE-co-suberoyl chloride) for Extrahepatic mRNA Delivery Following Intravenous Administration

Xueliang Yu, Shuai Liu, Qiang Cheng, Sang M. Lee, Tuo Wei, Di Zhang, Lukas Farbiak, Lindsay T. Johnson, Xu Wang and Daniel John Siegwart

Table S1. Raw data of in vitro screening of functional polyesters for luciferase mRNA delivery.

	PE4K																			
	A17				A3				A5				A6				A21			
SC2	4695	31449	21996	115198	7678	523	460	3264	0	−3	36	3	−23	−3	−23	10	43	50	40	66
SC3	3917	109028	283912	190542	473	136	270	2086	3	−9	26	236	−13	−19	10	130	253	226	90	213
SC4	7834	162401	344494	151908	280	146	173	8980	13	36	690	1937	7	7	−6	−6	56	606	216	96
SC5	30524	709836	209496	217245	113	170	256	3100	3	50	3908	1273	10	−19	−16	1323	463	14937	8289	20943
SC6	1335491	1600650	506464	425000	76	190	170	233	30	1433	54837	11518	13	27	760	69533	5796	61715	111820	45095
SC7	5347891	8567442	1814832	1056697	510	100	157	883	46	1210	90020	15157	13	23	15002	153478	2524	15025	9141	3574
SC8	3215210	11153	426267	130468	67	384	2634	2270	1240	2136	173859	210893	523	583	9948	31747	17851	55574	34361	93612
SC9	197807	15071	16497	13479	130	273	703	3701	940	1376	118793	248319	40	7768	7207	10005	1368494	1327292	664925	20353
SC10	32118	520379	209529	5128	67	67	80	93	520	9226	134075	218545	2910	2437	32941	10499	3559842	1686725	554024	35094
SC11	4558	13507	34362	25709	260	470	1180	107	506	863	20726	239476	3054	11027	6388	22724	2466850	1644258	908776	233777
SC12	1247	3170	14212	96726	286	66	93	109	413	3123	5258	61047	1153	620	1327	4371	1132445	1180560	483729	61400
SC14	35995	522077	3311	4655	316	66	53	116	46	336	9	196	123	1077	3197	10893	1367468	646917	760790	94148
SC16	2110055	2474115	823932	314166	56	1940	1583	266	23	79	113	706	323	2270	1297	1683	17979	1343	289	299
SC18	2400	4805	337	5923	886	423	1519	3194	10	73	86	396	6300	7370	1917	1283	8359	18458	3067	303
SC4-1	21095	7040	17037	54645	146	126	143	113	43	0	393	8582	0	20	33	100	193	196	199	139
SC5-1	11170	47348	149090	287449	3504	1036	160	470	40	43	2233	400	23	13	26	7264	26	130	116	63
SC8-1	6376900	2129110	2013409	892731	250	346	406	8015	943	6737	259225	4715	36	4845	21542	39679	180	940	326	126
SC8-Ph	13551	149094	332641	206320	1270	1570	290	1446	26	753	590	16	63	29	369	723	20	63	3	10
S0 6	78937	29810	11236	8409	186	286	360	1016	20	10	33	80	23	10	19	3274	190	520	6947	6666
S0 11	71844	66581	11320	8305	436	606	1406	3717	36	190	296	74799	40	26	143	956	180	66	80	163

	PE8K																			
	A17				A3				A5				A6				A21			
SC2	96	333	213	946	3	10	6	6	40	33	250	3767	40	23	10	7	3	1956	2407	943
SC3	3220	2316	3834	4455	13	6	26	10	46	93	533	11591	83	13	3	17	710	1750	6586	1413
SC4	2754	10229	22420	15379	10	19	6	6	83	133	1986	88417	17	13	20	13	1376	986	186	12280
SC5	3941	10172	30160	3277	3	13	6	0	46	533	24007	177279	10	7	17	40	1310	3550	8983	1003
SC6	8549	6336	22539	1119	13	−3	23	426	133	7819	157737	588267	17	7	20	33	1206	10402	3354	2874
SC7	25888	46849	11972	430	10	7	6	0	4325	5035	254553	27536	6	23	336	589	61842	30415	2580	123
SC8	14647	42109	13781	197	−3	0	6	1489	540	7993	24448	680	36	13	153	53	163065	9794	847	227
SC9	211244	375346	10046	177	−10	−13	110	0	50	37	540	1060	23	439	13	3	206611	284891	463	77
SC10	141031	356554	3884	200	−6	10	−7	29	93	2387	1273	463	966	339	36	23	6473	1897	157	153
SC11	62679	13962	31336	327	133	386	13	−3	93	243	6149	4091	209	769	19	339	414683	32692	680	130
SC12	472140	600513	52541	550	40	6	10	3	67	17	387	1483	470	207	123	87	1900	1256	149	30
SC14	435699	456355	14660	3908	3	−3	26	23	47	43	190	190	193	330	83	230	16623	156	746	566
SC16	269047	35597	16873	1583	160	33	43	30	37	83	53	30	330	393	47	37	1103	529	146	93
SC18	112550	23676	235783	70221	7537	5505	1203	613	67	17	27	80	10194	3707	523	77	49016	18447	459	96
SC4-1	6217	5575	8609	10949	13	3	7	3	30	10	780	28106	23	7	4	7	36	986	346	369
SC5-1	8914	5879	15085	6090	−3	6	7	1777	30	1240	74992	3040	47	7	4	70	7	53	133	516
SC8-1	6510	15520	22395	12959	0	7	680	13	743	3108	275006	1227	77	17	27	64	200	129	86	0
SC8-Ph	2296	4231	27901	83	13	3	10	3	323	29117	2983	5392	7	0	−3	10	29	10	13	−3
S0 6	426	153	276	330	3	0	−6	10	60	267	33	5095	10	3	13	17	10	16	76	909
S0 11	313	363	263	623	3	10	6	16	447	2647	1467	115639	7	7	3	17	66	39	10	6

	PE17K																			
	A17				A3				A5				A6				A21			
SC2	43	40	330	120	0	(3)	(0)	(6)	6	29	6	96	6	6	3	13	17	17	20	17
SC3	2250	10786	4368	73	46	109	(10)	(13)	3	3	20	5863	16	3	6	16	7	23	10	13
SC4	10003	47844	5098	103	2727	(3)	10	146	10	6	7	1793	56	16	6	9	30	7	20	27
SC5	107387	223981	200	147	70	6	(10)	226	26	19	2384	2110	16	19	7	6	0	17	17	17
SC6	79727	215751	110	100	(10)	(6)	20	(6)	6	123	33764	36	16	0	0	19	13	27	150	17
SC7	449687	163232	22475	149	10	207	140	407	13	26	1020	3	13	13	184	(6)	(3)	4	(9)	4
SC8	41459	109	43	83	117	143	237	10	17	333	366	13	0	7	3	0	(3)	4	7	(9)
SC9	109087	33456	119	103	147	7	7	23	10	43	290	23	4	17	10	7	7	540	(12)	(6)
SC10	50	56	33	59	23	20	17	80	33	113	330	13	30	4	7	10	0	(6)	(9)	7
SC11	15557	8636	43	39	1080	13	13	43	90	110	410	10	14	30	23	30	150	163	(9)	20
SC12	9884	39160	63	56	30	10	20	7	3	90	250	93	10	87	53	300	13	30	3	13
SC14	25347	31572	506	109	20	7	17	60	43	117	30	7	20	20	23	10	240	433	153	13
SC16	32039	2043	19	36	53	40	23	33	217	397	14	0	40	140	13	57	60	20	26	10
SC18	99	49	266	43	73	640	4334	443	20	7	10	17	7	243	130	160	350	206	20	36
SC4-1	17875	37822	4465	29	703	17	13	13	0	0	1	50	7	37	4	3	3	0	3	7
SC5-1	22668	75303	30726	869	10	3	0	(6)	7	10	3971	13	(3)	0	1	(3)	27	7	33	17
SC8-1	2117228	12085	416	203	3	40	266	17	7	(3)	2737	80	10	(3)	30	10	13	27	23	7
SC8-Ph	4067	73	5376	146	60	90	46	93	(3)	177	253	0	17	0	14	13	27	17	27	13
S0 6	133	129	776	823	123	70	93	1336	10	(3)	(3)	23	(3)	7	10	7	10	20	42	10
S0 11	206	179	96	56	40	113	10	27	37	43	37	67	0	10	(3)	7	6	15	10	15

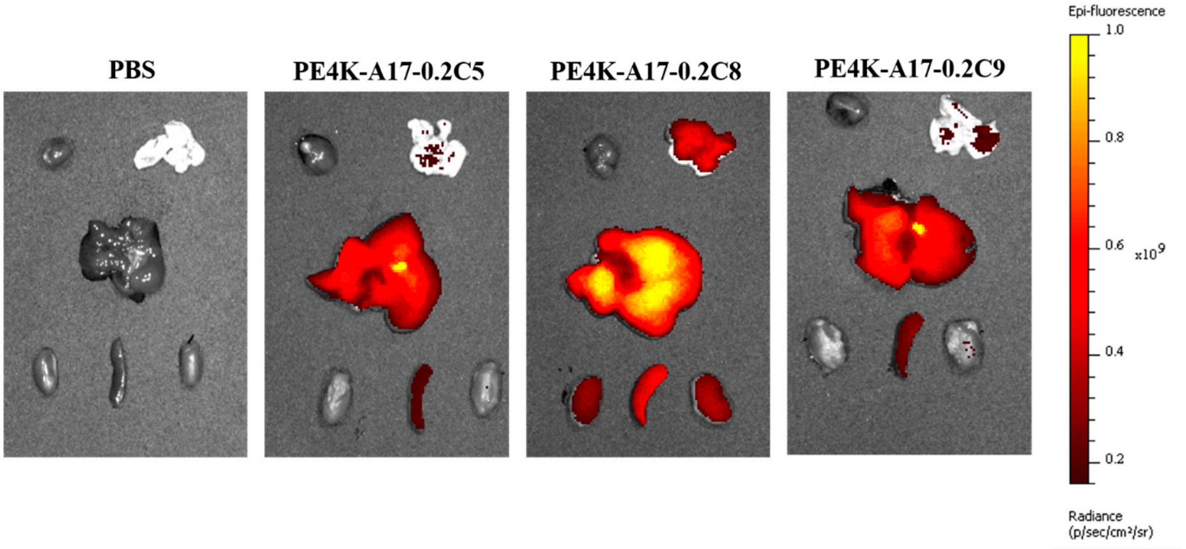

Figure S1. Imaging of mice organs after injection of Cy-5 mRNA loaded functional polyesters.

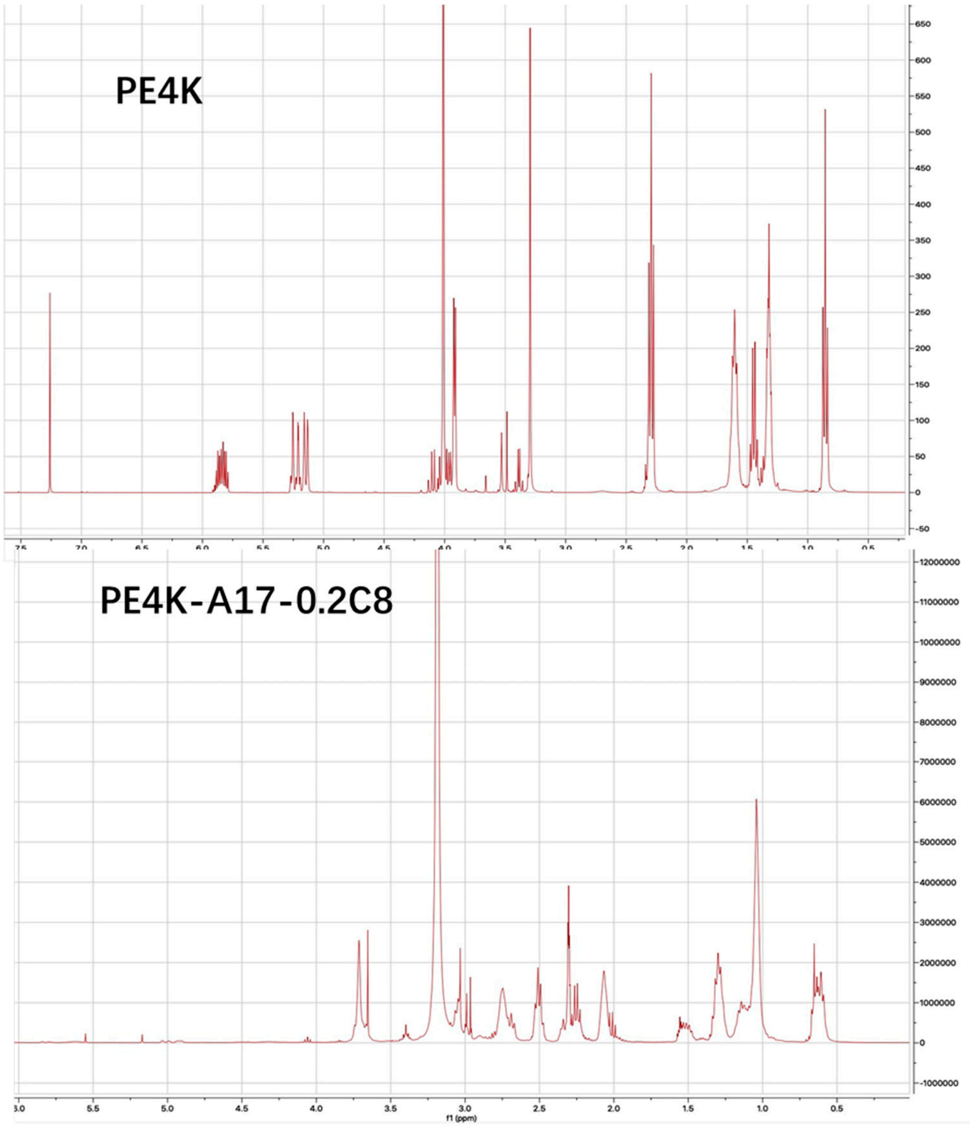

Figure S2. Selected NMR of polyester and functional polyester.

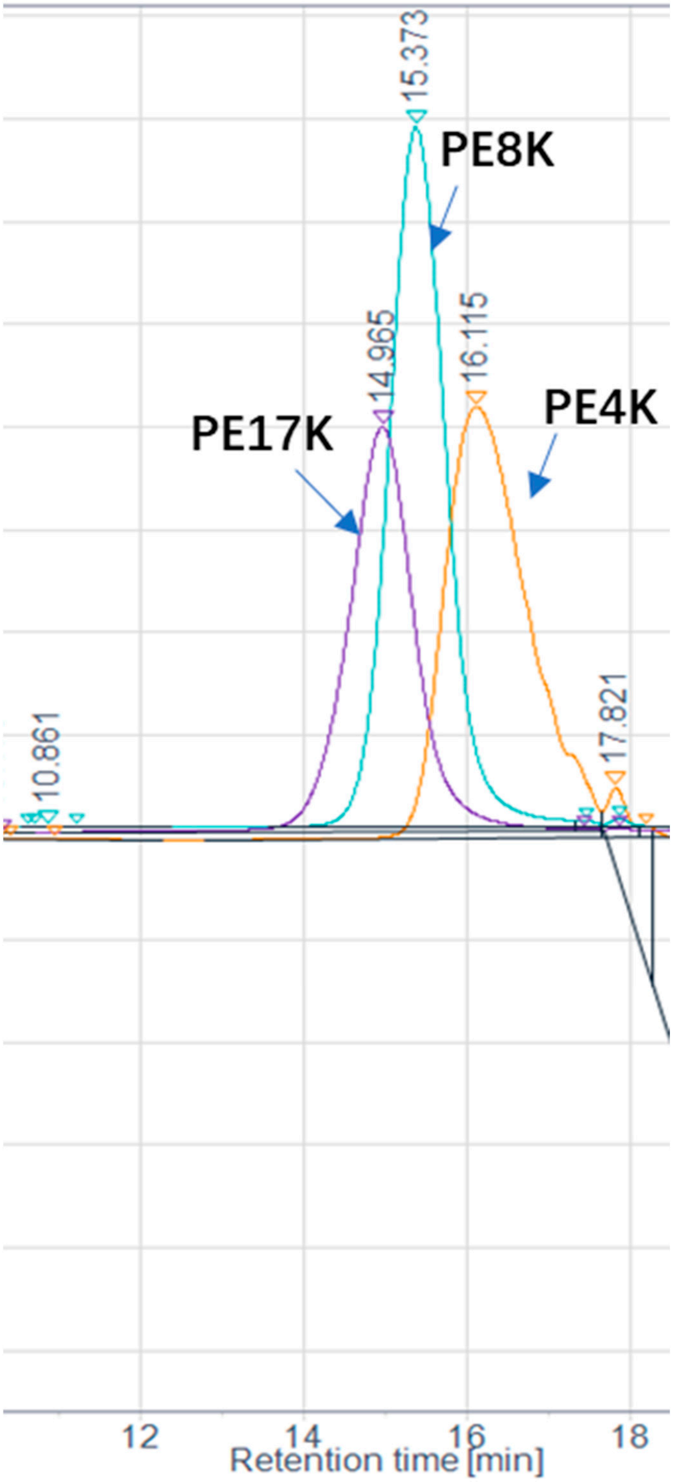

Figure S3. GPC (THF) curves of polyesters.
